# Association of* IGF-1 CA(n)* and* IGFBP3 rs2854746* Polymorphisms with Endometrial Polyp Risk

**DOI:** 10.1155/2018/8704346

**Published:** 2018-12-13

**Authors:** Pedro Leopoldo Silva Doria, Thomas Moscovitz, Marcos Tcherniakovsky, Cesar Eduardo Fernandes, Luciano Melo Pompei, Milton Wajman, Angela Van Nimwegen, Sergio Haimovich

**Affiliations:** ^1^Department of Obstetrics and Gynecology, Faculdade de Medicina do ABC, Santo Andre, SP, Brazil; ^2^Head of the Hysteroscopy Unit, Del Mar University Hospital, Barcelona, Spain; ^3^Head of Gynecology Ambulatory Surgery, Hillel Yaffe Medical Center/Technion-Israel Technology Institute, Hadera, Israel

## Abstract

**Introduction:**

Insulin-like growth factor 1* (IGF-1)* is a peptide growth factor that promotes cell proliferation and inhibits apoptosis. The bioavailability of* IGF-1* is regulated by the insulin-like growth factor binding protein 3* (IGFBP3).* Genetic variations influence the levels of* IGF-1* and* IGFBP3*. The purpose of this study was to examine the association of polymorphisms* IGF-1 CA(n)* and* IGFBP3 rs2854746* with risk of endometrial polyps.

**Materials and Methods:**

Case control observational study, composed of 104 women with antecedent of endometrial polyp (case group) and 81 postmenopausal women without antecedent of endometrial diseases (control group). Genotyping of* IGF-1 CA(n)* was performed by PCR and fragment analysis by capillary electrophoresis, and genotyping of* IGFBP3 rs2854746 *was performed by PCR-HRM. Odds ratios (ORs) and 95% confidence intervals (CIs) were calculated by logistic regression.

**Results:**

The genotype* IGF-1 CA(19)/CA(19)* was associated with an increased endometrial polyp risk (OR=2,57; IC 95%= 1,09 - 6,01); this was also found when combining it with* CA(>19)/CA(n)* genotypes (OR=2,18; IC 95%= 1,06-4,47). The* IGFBP3 rs2854746* analyses showed the* CG* genotype having a protective effect for endometrial polyp (OR=0,37; IC 95%= 0,19-0,73), fact also observed when grouping* CG* and* GG* carriers (OR=0,51; IC 95%= 0,28-0,93).

**Conclusion:**

The genotypes* CA(19)/CA(19)* and* CA(19)/CA(19)* +* CA(>19)/CA(n)* of the* IGF-1 CA(n)* may be considered a risk for endometrial polyp, whereas the genotypes* CG* and* CG* +* GG* of* IGFBP3 rs2854746* polymorphism have an inverse effect of endometrial polyp risk.

## 1. Introduction

Endometrial polyps are defined as nonmalignant, pedunculated, or sessile nodules composed of either functional or basal endometrium or a combination of the two [1]. The prevalence of endometrial polyps varies between 6% and 32%, depending on the definition of a polyp, the diagnostic method used (transvaginal sonography, hysteroscopy, and/or sonohysterography), and the population studied [2].

The pathogenesis of endometrial polyp is multifactorial, and imbalance between proliferation and apoptosis plays a pivotal role in the process. Cell proliferation and apoptosis in the endometrium are complex events involving several signaling pathways, including the insulin-like growth factor [3].

The* IGF* system includes insulin-like growth factors 1 and 2 (*IGF-1 and IGF-2*), their receptors (*IGF-1R* and* IGF-2R*), and six binding proteins (*IGFBP 1-6*). Both* IGF-1* and* IGFBP-3* are growth hormone dependent [4].

*IGFBP-3* is the major* IGF*-binding protein in serum and it serves as a reservoir for the* IGFs* in circulation [5–8].* IGFBP-3* carries* IGF-1* in circulation and directs it to target tissues, protects it from proteolytic degradation, and regulates its interaction with the* IGF-1R*. Additionally,* IGFBP-3* has its own* IGF*-independent apoptotic effects, mediated through a specific cell surface receptor [9].

Both* IGF-1* and* IGFBP-3* genes are polymorphic in human populations [10]. A polymorphism in the* IGF-I* gene has comprising a variable length of a* CA* repeat sequence* (IGF-1 CA*_*( n)*_) [11].

The* IGF* system is an important regulator of cell proliferation, differentiation, and apoptosis in various tissues. Several studies suggest that the imbalance between serum levels of* IGF-1* and* IGFBP3* could trigger abnormal cell proliferation, increasing the risk of neoplastic diseases [12].

The polymorphisms that alter gene expression or protein function may result in increased or decreased circulating levels of* IGF-1* and* IGFBP-3*, and therefore, influence the risk of endometrial disease [7].

The aim of the present study was to investigate the relationship between risk of endometrial polyp and genotypes of* IGF-1 CA*_*( n)*_ and* IGFBP-3 rs2854746* polymorphisms.

## 2. Material and Methods

### 2.1. Study Population

A hundred and eighty-five women were evaluated in this molecular epidemiological study. Women were divided into two groups: study and control groups. The study group consisted of 104 patients with previous history of hysteroscopic polypectomy that underwent surgery between 2012 and 2015. All of them were symptomatic (infertility, menorrhagia, or postmenopausal vaginal bleeding) before surgery and had confirmed endometrial polyp by histological examination. The control group consisted of 81 postmenopausal women without previous history of endometrial pathology and endometrial thickness less than 5 millimeters (transvaginal ultrasonography) that came to outpatient office for routine gynecologic visit.

Patients with a history of any cancer or tamoxifen were excluded from the study.

The fact that all patients in the control group are menopausal reinforces the influence of the polymorphism on the genesis of the polyp.

Women from both groups were asked to participate in the study and after their written informed consent, a short questionnaire and peripheral blood sample were obtained from all participants. The study was approved by the Research Ethics Committee of the Institution (CAAE 08657412.5.0000.0082).

### 2.2. Genotyping

DNA was extracted by standard protocols from peripheral blood as previously described [13]. Concentration and purity were verified by spectrophotometry (*NanoDrop®*,* USA*). Genotyping of the* IGF-1* microsatellite polymorphism (cysteine-alanine, or CA, repeat) was determined by PCR amplification of the polymorphic region followed by capillary electrophoresis analyses using the* ABI 3500 DNA Sequencer* (Applied Biosystems, Foster City, CA), as previously described [14]. PCR primers were forward 5′-GCTAGCCAGCTGGTGTTATT-3′ and Reverse: 5′-ACCACTCTGGGAGAAGGGTA-3′; the primer forward was 5′-labeled with a fluorescent dye (6-FAM). Fragment sizing was determined by Genescan analyses software (ABI Applied Biosystems). The fragments ranged in size from 174 to 202 base pairs, depending on the number of* CA* repeats. Representative homozygotes (18/18, 19/19, 20/20, and 21/21) were sequenced to determine* (CA)n* repeat number from base pair length. Quality control procedures were inclusion of positive and negative controls in each assay run; and 20 samples were repeated blindly to validate the genotyping procedures. The concordance for the blinded repeat samples was 100 %.

Genotyping of* rs2854746* polymorphism in* IGFBP3* was done by PCR-HRM (High Resolution Melting). PCR primers used to amplify the mutation were forward* 5*′*-CTGGGCCGCTGCGCTGACTCT-3*′ and reverse:* 5*′*-GCTCGCAGCGCACCACGGGAC-3*. PCR reaction contained 0,128mM of forward and reverse primers; 12,5ul of* Type-it®* HRM PCR kit* EVA GREEN®* and 2,0 *μ*l do genomic* DNA* (20ng). Total volume per reaction was 25*μ*l. Amplification was carried out at Rotor Gene 6000 (Qiagen,* USA*) using the following program: preincubation for 5 min at 95°C and amplification for 40 cycles of 30 s at 95°C, 30 s at 58,4°C and 45 s at 72°C, after which a high resolution melting curve was generated using the following protocol: 5 s at 95°C, 1 min at 60°C, followed by a gradual increase in temperature from 60°C to 97°C, using a ramp rate of 0.1°C per s, with one measurement per 2 s. Quality control included in all assay run included a previously sequenced homozygous* C* and* G* allele samples, heterozygous* CG* sample, and a negative sample; and 20 samples were repeated blindly to validate the genotyping procedures. The concordance for the blinded repeat samples was 98%.

### 2.3. Statistical Analysis

“Genotypic counts of controls were tested for Hardy–Weinberg equilibrium using* Chi*-square (*χ*^*2*^) test. Allele estimates were determined, as well as the frequencies of the most common alleles for gene* IGF-1* and* IGFBP3*. Linkage disequilibrium (LD) statistics were computed using Haploview 4.0. Logistic regression was performed with the presence of polyp as a dependent variable and polymorphisms as independent variables.” [15]. Age, sex, and body mass index were used as confounders. Odds ratios (ORs) and 95% confidence intervals (CI) were estimated for each polymorphism; reference categories were wild type. Gene-gene interactions were investigated by estimating models with two polymorphisms, one of each of the two genes. Likelihood ratio tests were conducted to compare a model with interaction effect between the two polymorphisms to a model without interaction term. Analysis of the data was performed using the software SPSS® for Windows version 17. All P values are two-sided; P values < 0.05 were considered to be statistically significant.

## 3. Results

There were 185 women included in this study. They were divided into two groups: control (n=81) and study (n=104). Personal and lifestyle characteristics of both groups are shown in Table 1. Significant differences between the groups were observed in three variables: age, prevalence of high blood pressure, and previous use of hormonal therapy. 46 patients (44%) of the polyp group are menopausal and 56% are in the reproductive period.

All* IGF-1* and* IGFBP3* alleles were in Hardy–Weinberg equilibrium in the control population (*IGF-1*: p = 0,48 and* IGFBP3*: p = 0,32).

There were seven different* IGF-1* alleles, ranging from 16 to 22* CA* repeats. The* IGF-1 CA(19)* was the most common allele in both groups (control: 51% and study: 56%), followed by* CA(20)* allele (control: 15% and study: 18%),* CA(18)* allele (control: 19% and study: 10%), and* CA(21)* allele (control: 9% and study: 11%). No other allele frequency exceeded 5% (data not shown). Overall, the* CA(19)/CA(19)* genotype was most common (control: 23,8% e study: 32%). Next most common genotypes were* CA(19)/CA(20)* (control: 22,5% e study: 17%),* CA(18)/CA(19)* (control: 16,3% e study: 11%), and* CA(19)/CA(21)* (control: 12,5% e study: 11%).* IGF-1 CA(n)* genotypes were grouped in three different ways as an attempt to identify the influence of allele length. Regression analysis showed that homozygous* CA(19)* genotype is associated with endometrial polyp risk (OR=2,57, IC 95%= 1,09-6,01, p=,02). Further analyses grouping homozygous 19* CA* genotype with genotypes with one allele longer than* CA(19)* also represented a risk for endometrial polyp,* CA(>19)/CA(n)* (OR= 2,181, IC 95%= 1,06-4,47, p =0,03) (Table 2).

For* IGFBP3*, the* C* allele had a frequency of control: 65% and study: 70%. The* G* allele frequency was 35% in control and 30% in the study group. The most common genotypes were* CG* in control group (48%) and* CC* in study group (57%). Regression analyses showed that* CG* genotype has a protective effect on endometrial polyp development (OR= 0,3; IC 95%= 0,195-0,730, p=0,003). Further analyses grouping* GG* and* CG* genotypes showed similar results (OR= 0,51; IC 95%= 0,284-0,937, p=0,029). (Table 3)

Interaction among* IGF-1 CA(n)* and* IGFBP3 rs2854746* were also investigated. The results showed that homozygous* CA(19)* + genotypes with one allele longer than* CA(19) + CC* genotype were significant associated with endometrial polyp risk (OR= 4,27; IC 95%= 1,64-11,09. p=0,002) (Table 4). Further analyses correcting the results for confounders (age, high blood pressure, and previous use of hormonal therapy) showed similar results (OR= 3,71; IC 95%= 1,38-10,0; p=0,009). The* CG* genotype appeared as a protective factor (OR= 0,16; IC 95%= 0,06-0,40; p=0,0001) (Table 5).

## 4. Discussion

Genetic polymorphisms are natural variations in the genomic DNA sequence present in more than 1% of the population.* IGF-1* plays an important role in the regulation of cell proliferation, differentiation, and apoptosis with a recognized effect on tumor growth [16].

The* IGF-1* gene is located on* chromosome 12 (12q 22–24.1*). It contains in the promoter region a microsatellite comprising a variable length of* CA* repeat sequence, which ranges from 10 to 24 [14].

The importance of* IGF-1 CA(n)* polymorphism relies on its association with* IGF-1* levels. This effect seems to be dependent on the number of* CA* repeats with higher circulating* IGF-1* levels for the* CA(19) *and* CA(20)* repeats alleles, while both alleles shorter than* CA(19)* and longer than* CA(20)* repeats seem to have lower circulating* IGF-1* levels [10, 17].

In the present study we found a statistically significant association between endometrial polyp risk and homozygous* CA(19)* genotype. Furthermore, grouping homozygous* CA(19)* genotype with genotypes with one allele longer than* CA(19)* also represented a risk for endometrial polyp. These results suggest that endometrial polyp risk is dependent on the number of* CA* repeats [7]. So, there is a lack of information regarding the effect of genetic factors on endometrial polyp risk. Therefore, our results give new insight over potential heritable factors associated with the development of endometrial polyp.

The underlying mechanisms by which some genotypes of* IGF-1 CA(n)* polymorphism increases the risk of endometrial polyp was not addressed in our study. Nevertheless, we speculates that* CA(19)* and* CA(>19)* alleles may be related to higher circulating* IGF-1* and, consequently, augment of endometrial proliferative activity. This hypothesis is based on other studies that found a relationship between high* IGF-1* levels and abnormal endometrial proliferative activity [18, 19].

The* IGFBP3* gene is located on* chromosome 7 (7p13)* and contains five exons. In exon 1 there is a nonsynonymous amino acid change, glycine to alanine. This change occurs at residue 32 in the protein structure, a region that has been shown, in fragment analyses, to contain a high-affinity binding region for* IGF-1* [20]. This polymorphism* (rs2854746)* may have an effect on the concentration of circulating* IGFBP3*, with* IGFBP3* levels increasing from* CC* →* GC* →* GG* in cancer-free individuals [21].

We found a statistically significant association between* IGFBP3 rs2854746* polymorphism and endometrial polyp risk, with* CG* genotype having a protective effect. Grouping* CG* and* GG* genotype carriers also showed significant inverse association with endometrial polyp risk. Explanation for this association stem from previously demonstrated relationship between* IGFBP3 rs2854746* polymorphism and* IGFBP3* levels, as the presence of* G* allele displayed higher* IGFBP3* levels when compared with* C* allele [21].

In our study, we also examined the interactions among variants of the two polymorphisms and endometrial polyp risk. The results showed that the association of* IGF-1* homozygous* CA(19)* or genotypes with one allele longer than* CA(19)* and* CC IGFBP3 rs2854746* represents a risk for endometrial polyp. This suggests that associations of endometrial polyp with* IGF *hormones may be causal and it is not restricted to one member of* IGF* family. Furthermore, the disequilibrium between* IGF-1* and* IGFBP3* levels could be the triggering factor for endometrial polyp development.

Some limitations of this study should be considered: (1) the number of participants per group did not allow us to verify the relationship between each homozygous genotype with endometrial polyp risk; (2) ethnicity of study population could not be determined due to Brazilian population admixture; and (3) significant differences in confounders (e.g., age, hormonal therapy use and high blood pressure) between control and study groups. Women from our study group were younger, with lower prevalence of high blood pressure and hormonal therapy use than women from control group. However, it should be noted that results from multiple logistic regression showed no influence of high blood pressure on the association between variants of the studied polymorphisms and endometrial polyp risk. The strength of our study is that controls were known to be polyp-free and with no history of endometrial diseases at the time of blood sampling. Our decision to select postmenopausal women as control group was based on their lifetime exposure to risk factors for endometrial diseases without developing them. We speculate that women with this profile might have some kind of protection against endometrial polyp risk factors.

The multiple logistic regressions showed protective influence of the advancement of age with endometrial polyp, because our control patients were all menopausal, and therefore this may have interfered with this result. Something similar occurs with the use of hormone replacement therapy, where the majority of patients who used the therapy were from the control group, and this made the therapy a protective effect in relation to the endometrial polyp.

The strength of our study is that controls were known to be polyp-free and with no history of endometrial diseases at the time of blood sampling. Our decision to select postmenopausal women as control group was based on their lifetime exposure to risk factors for endometrial diseases without developing them. We speculate that women with this profile might have some kind of protection against endometrial polyp risk factors.

Our study reinforces the importance of polymorphism in the genesis of the endometrial polyp and genetic variability gains more force as an important risk factor. The clinical importance of this study is that with it we can say that some polymorphisms are considered risk factors for endometrial polyp.

The results suggest that some genotypes of the IGF-1 CA(n) polymorphism have a risk ratio for endometrial polyp. However, some genotypes of the IGFBP3 polymorphism rs2854746 are inversely related to endometrial polyp. Therefore, it is possible to consider them as risk or protection factors, according to the genotype expression in question.

## Figures and Tables

**Table 1:** Epidemiological characterization of patients with arterial hypertension, use of hormone replacement therapy (HRT), age group, diabetes, and evaluation of body mass index (BMI).

	**Control**	**Polyp**	**O.R**	**95**%** C.I.**	**P-Value**
**Arterial hypertension**					

no	44(56%)	74(71%)			

yes	35(44%)	30(29%)	0,509	(0,275 -0,941)	0,031

no answer	2	0			

**HRT**					

no	44(56%)	89(87%)			

yes	34(44%)	13(13%)	0,189	(0,090 -0,393)	<0,001

no answer	3	2			

**Age range**					

29-39	3 (4%)	36(35%)			

40-49	8 (10%)	22(21%)	0,229	(0,054 -0,956)	0,043

50-59	52 (65%)	18(17%)	0,028	(0,007 -0,105)	<0,001

60-80	17 (21%)	28(27%)	0,137	(0,036 -0,515)	0,003

no answer	1	0			

**Diabetes**					

no	72 (92%)	96 (92%)			

yes	6 (8%)	8 (8%)	0,980		

no answer	2	0			

**Body Mass Index**					

normal	30 (39%)	36(37%)			

overweight	25 (33%)	35(35%)			

obese	21 (28%)	27(28%)	0,912		

no answer	5	6			

**Table 2:** Characterization of the sample for IGF-1 genotyping.

	**Control**	**Polyp**	**O.R**	**95**%** C.I.**	**P-Value**
**Grouping 1**					

CA(19)/CA(19)	19 (23%)	34 (33%)			

CA(19)/CA(n)	44 (54%)	49 (47%)			0,382

CA(n)/CA(n)	18 (22%)	21 (20%)			

**Grouping 2**					

CA(19)/CA(19)	19 (23%)	34 (33%)	2,57	(1,09-6,01)	0,024

CA(19)/CA(<19) + CA(<19)/CA(<19)	23 (28%)	16 (15%)	1		

CA(>19)/CA(n)	39 (48%)	54 (52%)	1,99	(0,93-4,25)	0,076

**Grouping 3**					

CA(19)/CA(<19) + CA(<19)/CA(<19)	23 (28%)	16 (15%)	1		

CA(19)/CA(19) + CA(>19)/CA(n)	58 (72%)	88 (85%)	2,18	(1,06-4,47)	0,033

**Total**	81	104			

A: alanine.

C: cysteine.

CA: allele cysteine-alanine.

**Table 3:** Characterization of the sample for IGFBP3 (rs2854746) genotyping.

**IGFBP3 rs2854746**	**Control**	**Polyp**	**O.R**	**95**%** C.I.**	**P-Value**
CC	32 (41%)	58 (57%)	1		

CG	38 (48%)	26 (26%)	0,37	0,19-0,73	0,003

GG	9 (11%)	18 (18%)	1,10	0,44-2,73	0,831

GG + CG	47 (59%)	44 (43%)	0,51	0,28-0,93	0,029

No answer	2	2			

C: cysteine.

G: glycine.

CC: allele cysteine-cysteine.

GG: allele glycine-glycine.

CG: allele cysteine-glycine.

**Table 4:** Results of interactions between IGF-1 CA(n) and IGFBP3 rs2854746.

**Interaction IGFBP3 e IGF1**	**Control**	**Polyp**	**O.R**	**95**%** C.I.**	**P-Value**
**CA(<19)/CA(n) + CC**	16 (20,3%)	11 (10,8%)			

**CA(<19)/CA(n) + CG**	5 (6,3%)	2 (2%)	0,581	0,09-3,55	0,557

**CA(<19)/CA(n) + GG**	2 (2,5%)	3 (2,9%)	2,181	0,31-15,28	0,432

**CA(19)/CA(19)+ CA(>19)/CA(n) + GG**	7 (8,9%)	15 (14,7%)	3,116	0,95-10,15	0,059

**CA(19)/CA(19)+ CA(>19)/CA(n) + CC**	16 (20,3%)	47 (46,1%)	4,272	1,64-11,09	0,002

**CA(19)/CA(19)+ CA(>19)/CA(n) + CG**	33 (41,8%)	24 (23,5%)	1,057	0,41-2,68	0,905

**No answer**	2	2			

**Total**	81	104			

**Table 5:** Multivariate logistic regression between the IGF-1 CA(n) and IGFBP3 rs2854746 polymorphisms adjusted for hypertension, age range, and hormone replacement therapy (HRT) use.

	**OR**	**95**%** C.I.**	**P-Value**
**HRT**			

não	1		

Sim	0,2462	(0,096-0,631)	0,003

**Arterial hypertension**			

não	1		

sim	0,8201	(0,341-1,968)	0,657

**Age range**			

29-39	1		

40-49	0,2012	(0,042-0,946)	0,042

50-59	0,0326	(0,007-0,143)	0,005

60-80	0,1387	(0,029-0,647)	0,012

**IGF-1 CA(19)**			

CA(<19)/CA(n)	1		

CA(19)/CA(19) + CA(>19)/CA(n)	3,7191	(1,380-10,020)	0,009

**IGFBP3**			

CC	1		

CG	0,1607	(0,063-0,409)	0,001

GG	0,6648	(0,192-2,298)	0,518

## Data Availability

The data used to support the findings of this study are available from the corresponding author upon request.

## References

[1] Mazur M. T., Kurman R. J. (1995). *Diagnosis of Endometrial Biopsies and Curettings*.

[2] Dreisler E., Stampe Sorensen S., Ibsen P. H., Lose G. (2009). Prevalence of endometrial polyps and abnormal uterine bleeding in a Danish population aged 20–74 years. *Ultrasound in Obstetrics & Gynecology*.

[3] Arends M. J. (1999). Apoptosis in the endometrium. *Histopathology*.

[4] Yu H., Rohan T. (2000). Role of the insulin-like growth factor family in cancer development and progression. *Journal of the National Cancer Institute*.

[5] Pollak M. (2000). Insulin-like growth factor physiology and cancer risk. *European Journal of Cancer*.

[6] Vaessen N., Heutink P., Janssen J. A. (2001). A polymorphism in the gene for IGF-I: functional properties and risk for type 2 diabetes and myocardial infarction. *Diabetes*.

[7] McGrath M., Lee I.-M., Buring J., De Vivo I. (2011). Common genetic variation within IGFI, IGFII, IGFBP-1, and IGFBP-3 and endometrial cancer risk. *Gynecologic Oncology*.

[8] Pavelić J., Radaković B., Pavelić K. (2007). Insulin-like growth factor 2 and its receptors (IGF 1R and IGF 2R/mannose 6-phosphate) in endometrial adenocarcinoma. *Gynecologic Oncology*.

[9] Marshman E., Streuli C. H. (2002). Insulin-like growth factors and insulin-like growth factor binding proteins in mammary gland function. *Breast Cancer Research*.

[10] Deal C., Ma J., Wilkin F. (2001). Novel promoter polymorphism in insulin-like growth factor-binding protein-3: Correlation with serum levels and interaction with known regulators. *The Journal of Clinical Endocrinology & Metabolism*.

[11] Weber J. L., May P. E. (1989). Abundant class of human DNA polymorphisms which can be typed using the polymerase chain reaction. *American Journal of Human Genetics*.

[12] Ma J., Pollak M. N., Giovannucci E. (1999). Prospective study of 356 colorectal cáncer risk in men and plasma levels of insulin-like growth factos IGF-1 and IGF binding protein-3. *JNCI Journal of the National Cancer Institute*.

[13] Embrapa. Fundamentos teórico-práticos e protocolos de extração e de amplificação de DNA por meio da técnica de reação em cadeia da polimerase. São Carlos. Jun 2007. Avaliable from http://www.cppse.embrapa.br/servicos/publicacaogratuita/e-books/LVFundDNA Cited 18 January 2018

[14] Keku T. O., Vidal A., Oliver S. (2012). Genetic variants in IGF-I, IGF-II, IGFBP-3, and adiponectin genes and colon cancer risk in African Americans and Whites. *Cancer Causes & Control*.

[15] Feik E., Baierl A., Hieger B. (2010). Association of IGF1 and IGFBP3 polymorphisms with colorectal polyps and colorectal cancer risk. *Cancer Causes & Control*.

[16] Harrela M., Koistinen H., Kaprio J. (1996). Genetic and environmental components of interindividual variation in circulating levels of IGF-I, IGF-II, IGFBP-1, and IGFBP-3. *The Journal of Clinical Investigation*.

[17] Rietveld I., Janssen J. A. M. J. L., Van Rossum E. F. C. (2004). A polymorphic CA in the IGF-I gene is associated with gender-specific differences in body height, but has no effect on the secular trend in body height. *Clinical Endocrinology*.

[18] Rutanen E.-M. (2000). Insulin-like growth factors and insulin-like growth factor binding proteins in the endometrium. Effect of intrauterine levonorgestrel delivery. *Human Reproduction*.

[19] Nagamani M., Stuart C. A., Dunhardt P. A., Doherty M. G. (1991). Specific binding sites for insulin and insulin-like growth factor I in human endometrial cancer. *American Journal of Obstetrics & Gynecology*.

[20] Zou T., Fleisher A. S., Kong D. (1998). Sequence alterations of insulin-like growth factor binding protein 3 in neoplastic and normal gastrointestinal tissues. *Cancer Research*.

[21] Morimoto L. M., Newcomb P. A., White E., Bigler J., Potter J. D. (2005). Variation in plasma insulin-like growth factor-1 and insulin-like growth factor binding protein-3: Genetic factors. *Cancer Epidemiology, Biomarkers & Prevention*.

